# *Acrostichum*, a Pioneering Fern of Floodplain Areas from the Late Oligocene Sariñena Formation of the Iberian Peninsula

**DOI:** 10.1371/journal.pone.0162334

**Published:** 2016-09-15

**Authors:** Rafael Moreno-Domínguez, Borja Cascales-Miñana, Javier Ferrer, José B. Diez

**Affiliations:** 1 Área de Paleontología, Departamento de Ciencias de la Tierra, Universidad de Zaragoza, 50009 Zaragoza, España; 2 PPP, Department of Geology, University of Liege, Allée du 6 Août, B18 Sart Tilman, B4000 Liege, Belgium; 3 Dpto. Geociencias Marinas y Ordenación del Territorio, Universidad de Vigo, Campus Lagoas-Marcosende, 36200 Vigo Pontevedra, España; Institute of Botany, CHINA

## Abstract

*Acrostichum* is considered today an opportunistic fern in disturbed areas, which indicates the first stages of colonisation of such zones. However, in the fossil record, *Acrostichum* appears related to fluvio-lacustrine environments, freshwater marshes and mangrove deposits. We report here for first time fossil evidence of *Acrostichum* that reveals a pioneering behaviour of this fern in the colonisation of perturbed communities in Europe, which corroborates previous assumptions about the paleobiology of *Acrostichum*. Plant remains were collected from the Chattian (late Oligocene) La Val fossil site (Estadilla, Huesca, northeastern Spain) belonging to the Sariñena Formation, which mainly embraces crevasse splays, levees and floodplain deposits. Evidence shows that *Acrostichum* grew within the levee’s vegetal community or close to/on the river banks as well as on floodplain areas and closer to/on the shores of ephemeral ponds. But most importantly, the observed co-existence of *Equisetum* and *Acrostichum* remains in the same beds indicates that such strata represent short-lived inundated terrains, e.g., floodplains where the water table was temporarily stagnant. Evidence shows wetland environments dominated by pioneering taxa, implying a pioneering role for *Acrostichum* during the late Oligocene in the Iberian Peninsula.

## Introduction

*Acrostichum* Linnaeus is a rhizomatous fern of the Pteridaceae Kirchner family [1]. This plant is a common part of the understory of mangrove backwaters and is the only fern that can grow in brackish water [2]. In particular, the most characteristic habitat for *Acrostichum* is inshore marsh areas that receive some saline water from high tides and some fresh water from inflowing streams [3]. It grows in groups, sometimes gregariously colonising an area, and it is characterised by a pantropical distribution [2,4]. It usually develops in organic and clay-rich soils of high salinity, with pH acidic to neutral [5,6]. *Acrostichum* is one of the few ferns characteristic of mangrove areas and is often described as “mangrove fern” [2]. *Acrostichum* can however also live along river margins inland at great distance from the coastline [7–9], in shallow soil on rock ledges, in inland freshwater swamps [3,10,11], and in inland springs, which may act as a substitute source of minerals and salt. *Acrostichum* can also grow at quite high altitudes (e.g., at 1158 m at Wau, Morobe, New Guinea [4]).

*Acrostichum* colonises different habitats of current vegetation due to its ability for adaptation to disturbed areas devoid of local vegetation [2,11–13]. *Acrostichum* survives in a wide range of soil salinity and requires full sun exposure for a fast and maximum development [14]. This full sun exposure allows *Acrostichum* to rapidly exploit disturbed areas where the trees are gone or non-existent. *Acrostichum* is also considered as an opportunistic fern in altered estuarine environments or cleared areas of the mangrove [2,5]. Indeed, the fern is known in some countries as a “vegetable pest” because its profuse growth can impede the regeneration of mangrove trees [14,15]. Moreover, the fossil record shows *Acrostichum* as a pioneering plant occurring in coastal and freshwater lakes, marsh, [16–18], and fluvio-lacustrine environments [6,11,13,16,18–20]. Other authors [5,21–24] have inferred their existence in paleomangrove swamps and brackish water areas with tidal influence.

The pioneering nature of fossil *Acrostichum* has barely been addressed in the literature. This genus typically receives little attention in the study of fossil floras due to its rare occurrence rate, as well as the poorly preserved state of collected evidence [6,11,13]. However, *Acrostichum* is an excellent indicator of changing environmental conditions [5,11,15,23] and therefore a key element in understanding the Cenozoic floras with regard to depositional environment and paleoecology. We present herein new fossil remains of *Acrostichum* collected from the La Val fossil site (Huesca Province, Spain). This study aims to: (1) describe the sedimentological setting and paleoecological conditions of the fossil assemblage; (2) compare these findings to similar current and ancient environmental conditions where this fern occurs; and (3) provide new insights into the paleoecological role played by *Acrostichum* during the late Oligocene in Europe.

## Location and Geological Settings

**Fig 1:**
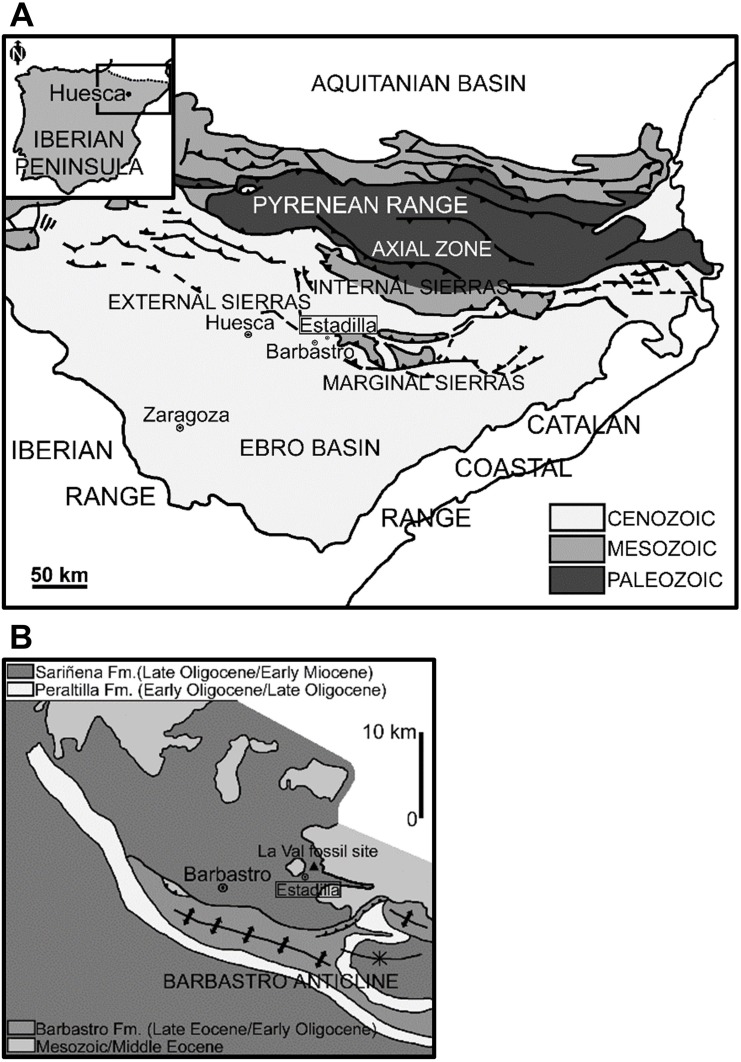
Localization and geological/geographical setting context of the La Val fossil site (Huesca Province, northeastern Spain). (A) General map showing the localization of the fossil assemblage in the Iberian Peninsula. Modified from [26] for illustrative purposes only. Common elements reprinted with permission from Elsevier, original copyright 2005. (B) High resolution map showing the fossil assemblage. Modified from [27] for illustrative purposes only. Common elements reprinted with permission from Geological Society of Spain, original copyright 1998.

The fossil site of La Val (42°3’47.62”N, 0°15’3.34”E) is located in the La Val ravine, one kilometre north of Estadilla (Huesca Province, Spain, Fig 1A). The outcrop is located in the Marginal Sierras at the tip of the South Pyrenean Central Unit (Pyrenean Range), very close to the eastern part of the northern flank of the Barbastro anticline in the northeast zone of the central sector of the Ebro Basin (Fig 1B). The Pyrenean Range is an orogen that resulted from a collision between the Eurasian and Iberian plates, and the Ebro Basin is the southern foreland basin of the Pyrenean Range [25–27]. This basin was endorheic and isolated from marine influence from the late Eocene to the late Miocene [27]. The La Val fossil site occurs in the Sariñena Formation, which is mainly situated in the central area of the northern Ebro Basin [25], and consists of a series of continental conglomerates, sandstone and mudstone beds. These terrigenous rocks are interpreted as fluvial and alluvial-fan deposits (e.g., [27]). The Sariñena deposits are subdivided into two tectosedimentary units (TSUs, [28]); corresponding to the units T4 and T5 defined for the Ebro Basin (see [25] for references). Their boundaries are dated using microvertebrate fossil evidence (see [26,27] for references). Unit T4 (Chattian-Aquitanian) includes the lower and middle part of the Sariñena Formation, whereas Unit T5 (Aquitanian-Burdigalian) only includes its upper part. From a stratigraphical viewpoint, La Val is situated in the lower part of this formation. The La Val fossil site is late Oligocene (Chattian) in age.

## Materials and Methods

The fossil collection consists of 58 specimens. This collection is housed at the Museum of Natural Sciences of the University of Zaragoza (Zaragoza, Spain). All necessary permits were obtained from the General Direction of Cultural Patrimony of Aragon, Spain (permit numbers: 047/2012, 047/12-2013, 047/12-13-2014 and 047/12/13/14/2015). The complete list of repository numbers is the following: EMPZ 2016/11-LV3-118-1A/1B, EMPZ 2016/11-LV3-119-1, EMPZ 2016/11-LV5-33-1A/1B, EMPZ 2016/11-LV5-34-1, EMPZ 2016/11-LV5-35-1, EMPZ 2016/11-LV5-36-1, EMPZ 2016/11-LV5-37-1, EMPZ 2016/11-LV5-38-1, EMPZ 2016/11-LV5-39-1, EMPZ 2016/11-LV5-40-1, EMPZ 2016/11-LV6-84-1A/1B, EMPZ 2016/11-LVNH-9-1A/1B, EMPZ 2016/11-LVNH-12-1, EMPZ 2016/11-LVNH-12-2, EMPZ 2016/11-LVNH-21-1A/1B, EMPZ 2016/11-LVNH-21-2A/2B, EMPZ 2016/11-LVNH-62-1, EMPZ 2016/11-LVNH-66-1, EMPZ 2016/11-LVNH-73-1, EMPZ 2016/11-LVNH-74-1, EMPZ 2016/11-LVNH-76-3, EMPZ 2016/11-LVNH-78-1, EMPZ 2016/11-LVNH-79-1, EMPZ 2016/11-LVNH-80-1, EMPZ 2016/11-LVNH-81-1A/1B, EMPZ 2016/11-LVNH-82-2, EMPZ 2016/11-LVNH-84-1, EMPZ 2016/11-LVNH-86-1, EMPZ 2016/11-LVNH-86-2A/2B, EMPZ 2016/11-LVNH-87-1, EMPZ 2016/11-LVNH-88-1, EMPZ 2016/11-LVNH-89-1, EMPZ 2016/11-LVNH-89-2, EMPZ 2016/11-LVNH-91-1A/1B, EMPZ 2016/11-LVNH-91-2, EMPZ 2016/11-LVNH-93-1A/1B, EMPZ 2016/11-LVNH-93-2A/2B, EMPZ 2016/11-LVNH-95-1, EMPZ 2016/11-LVNH-96-1, EMPZ 2016/11-LVNH-98-1, EMPZ 2016/11-LVNH2-3-1A/1B, EMPZ 2016/11-LVNH2-4-1, EMPZ 2016/11-LVNH2-5-1A/1B, EMPZ 2016/11-LVNH2-5-2, EMPZ 2016/11-LVNH2-8-1, EMPZ 2016/11-LVNH2-9-1, EMPZ 2016/11-LVNH2-12-1, EMPZ 2016/11-LVNH2-13-1, EMPZ 2016/11-LVNH2-14-1, EMPZ 2016/11-LVNH2-14-3, EMPZ 2016/11-LVNH2-15-2, EMPZ 2016/11-LVNH2-16-1, EMPZ 2016/11-LVNH2-17-1, EMPZ 2016/11-LVNH2-17-2, EMPZ 2016/11-LVNH2-18-1A/1B, EMPZ 2016/11-LVNH2-19-1A/1B, EMPZ 2016/11-LVNH2-19-2, EMPZ 2016/11-LVNH2-20-1A/1B.

**Fig 2:**
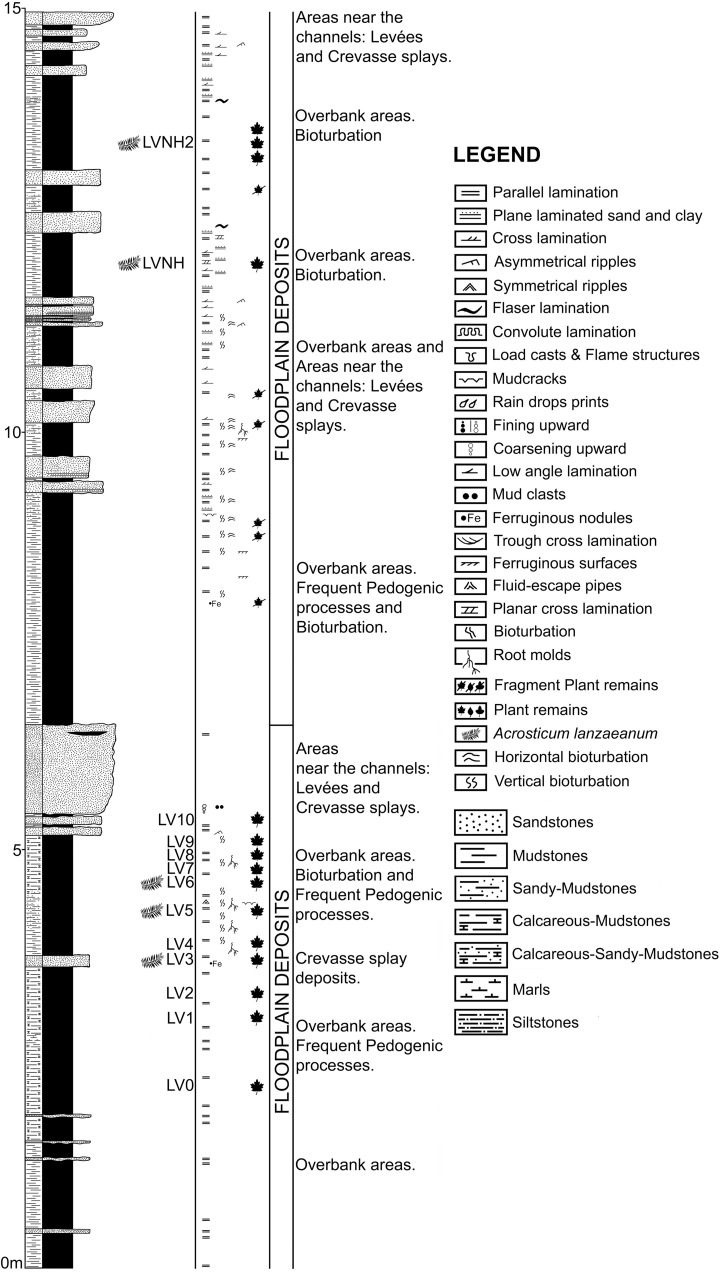
Stratigraphic profile of the La Val fossil site. Figure shows the location of collected samples, and the levels with plant megafossil remains of *Acrostichum*. A short description about the sedimentary environment is also supplied.

Plant remains are preserved as impressions and/or compressions. Each specimen was examined with a Nikon SMZ-2 stereo microscope and all photographs were taken with a Nikon D-90 camera fitted with an AF-S Micro Nikon 60 mm macro lens. A calliper and a ruler were used to measure the dimensions of the fossils; reported measurements of the length and width of specimens are based on the mean of measurements taken from 29 of the 58 (S1 Table). Regarding their taxonomy, the classification used is the one proposed for ferns by Smith et al. [1]. The distribution of specimens per sampled level is as follows: 2 from LV3, 8 from LV5, 1 from LV6, 29 from LVNH and 18 from LVNH2 (Fig 2). Inventory nomenclature: the initials “LV” indicates the fossil locality “La Val”, “NH” refers to *Nivel Helechos* or fern level in English, and the number indicates the stratigraphic level. For instance: LV3 indicates La Val at level 3 (stratigraphic level); LVNH2 indicates La Val at NH2 (fern level two).

## Results

### Depositional environment

**Fig 3:**
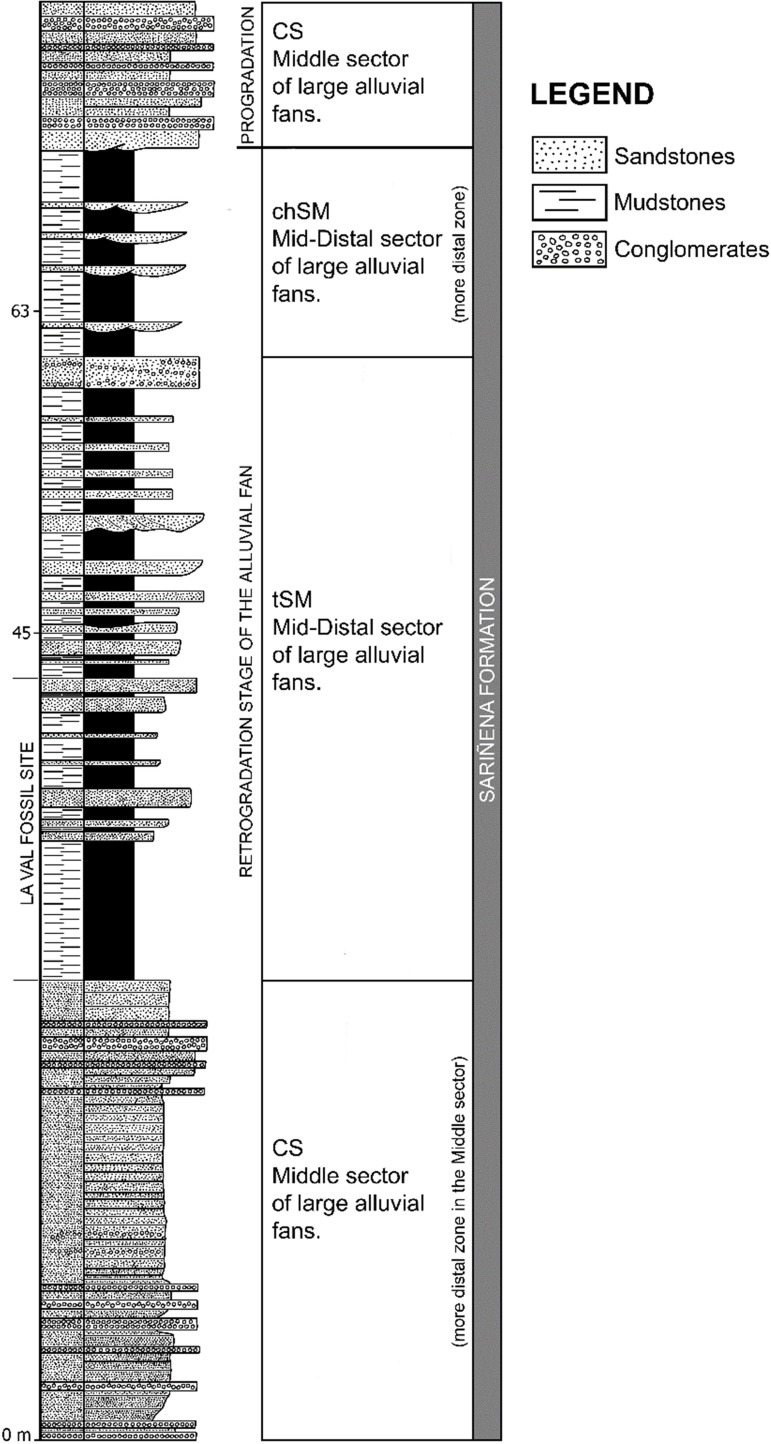
General lithostratigraphical profile of the fan where the La Val site is located. The profile shows the different lithofacies associations.

**Fig 4:**
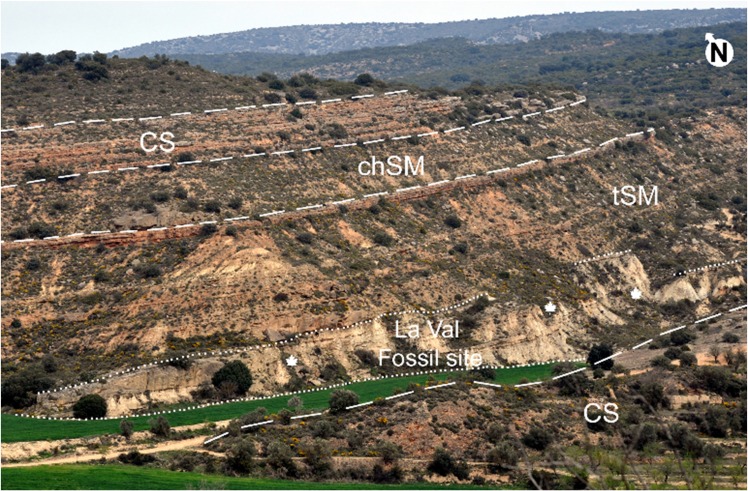
General view of the lithofacies associations present in the La Val ravine. Abbreviations: CS, Conglomerates and Sandstones; chSM, channelled Sandstones and Mudstones; tSM, tabular Sandstones and Mudstones. See Ref. 26 for details.

Three different lithofacies associations were identified in the study area (Figs 3 and 4). These facies were originally defined by Luzón [26,27] in the northern margin of the Ebro Basin using as criteria the percentage of each represented lithology, bed shapes, texture and sedimentary structures. Overall, all the facies and sediments are mainly associated with meandering river deposits.

Firstly, the CS (Conglomerates and Sandstones *sensu* Luzón [26]) facies are formed by grey conglomerates and brown sandstones. The thick-bedded tabular conglomerates (up to 70 cm in thickness) have clast-supported textures with a matrix of coarse to medium-fine sand, and rounded and well-sorted calcareous and quarzitic pebbles. Tabular beds (4–74 cm in thickness) of medium to fine, sometimes coarse-grained sandstones are present. These beds exhibit horizontal lamination, trough cross-stratification and current ripples. These facies are interpreted as braided streams with longitudinal and transverse bars, with overbank areas, which become more stable downstream.

Secondly, the tSM facies (tabular Sandstones and Mudstones *sensu* Luzón [26]) comprise grey, orange or yellow sandstones and grey or brown mudstones. Coarse to fine-grained sandstones also appear, sometimes with lags of mudstones. These deposits appear to be disposed in tabular bodies (up to 72 cm in thickness) often with channelled bases. They exhibit horizontal lamination, trough and planar cross-stratification and asimetric ripples. Mudstones are laminated, sometimes massive, and they are disposed in tabular beds (up to one meter in thickness). These mudstones exhibit horizontal and heterolitic lamination, bioturbation, roots and numerous vegetable remains. Also, beds of conglomerates up to 3 cm in thickness sometimes exit.

Finally, the chSM facies (channelled Sandstones and Mudstones *sensu* Luzón [26]) are formed of grey or orange sandstones and brown mudstones. Medium-grained sandstones appear to be disposed in tabular beds with channelled bases (up to 40 cm in thickness). They show horizontal lamination, trough and planar cross-stratification and ripples. Mudstones are laminated or massive, and disposed in tabular beds (10–50 cm in thickness) with horizontal lamination. Both tSM and chSM facies are characteristic of stable floodplains crossed by rectilineous and winding watercourses; levees and crevasses have developed in the channels adjacent to overbank areas. These deposits were affected by pedogenic processes (e.g., mottling, fossilised roots, precipitating calcium carbonate as small soil nodules), which also occurred in many ponds with carbonate deposits in interchannel areas.

#### La Val fossil site

The stratigraphic profile of the La Val fossil site (Fig 2) displays both sandstone and mudstone beds. Medium to fine, sometimes coarse-grained sandstones are present, generally disposed in tabular beds (up to 110 cm in thickness) and exhibit horizontal lamination, ripples, through and planar cross-stratification, and bioturbation. Interestingly, the fossil leaves and small woody fragments mainly recovered in these sediments. Mudstones are laminated to massive; they are disposed in tabular beds (1–87 cm in thickness) and show numerous sedimentary structures (e.g., roots or bioturbation, see S1 and S2 Figs for details) and frequent fossil plant remains (Fig 2). The La Val fossil site belongs to the tSM lithofacies. Sandstones correspond to levees and crevasse splay deposits and were deposited in areas very close to watercourses. The levees were sediment banks at the channel edge, whereas the crevasse splays were low cones of sediment. Moreover, mudstone beds are interpreted as floodplain or overbank deposits and were deposited not only in interchannel areas, but also in areas beyond the river channels that received water only when the river was in flood [29]. Most of these deposits contain little organic matter; indicative of a well oxygenated and drained environment where the efficient removal of organic matter was enabled by alkaline ground waters [30]. These deposits only show small rooting systems and the density of these rooting systems indicates that vegetation was not very abundant (e.g. S2G Fig). These root traces also vary from shallowly to deeply penetrating, suggesting a fluctuating water table [30].

#### Systematic Paleobotany

Class Polypodiopsida Cronquist, Takhtajan and Zimmermann

Order Polypodiales Link

Family Pteridaceae Kirchner

Genus ***Acrostichum*** Linnaeus

***Acrostichum lanzaeanum*** (Visiani) Reid and Chandler

(Figs 5 and 6)

**Fig 5:**
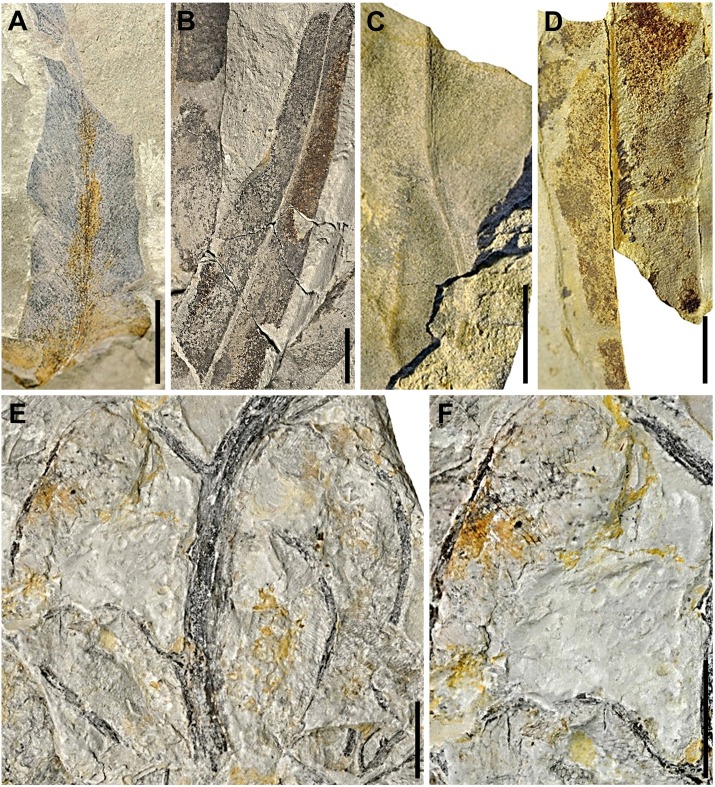
Plant megafossil remains of *Acrostichum lanzaeanum* discovered at the La Val fossil site (I). (A) EMPZ 2016/11-LV6-84-1B, pinna fragment from level LV6. (B) EMPZ 2016/11-LV5-40-1, pinnae from level LV5. (C) EMPZ 2016/11-LV3-118-1A, pinna fragment from level LV3. (D) EMPZ 2016/11-LV5-36-1, pinna fragment from level LV5. (E, F) EMPZ 2016/11-LVNH-12-2, frond fragments from level LVNH. Scale bars = 1 cm.

**Fig 6:**
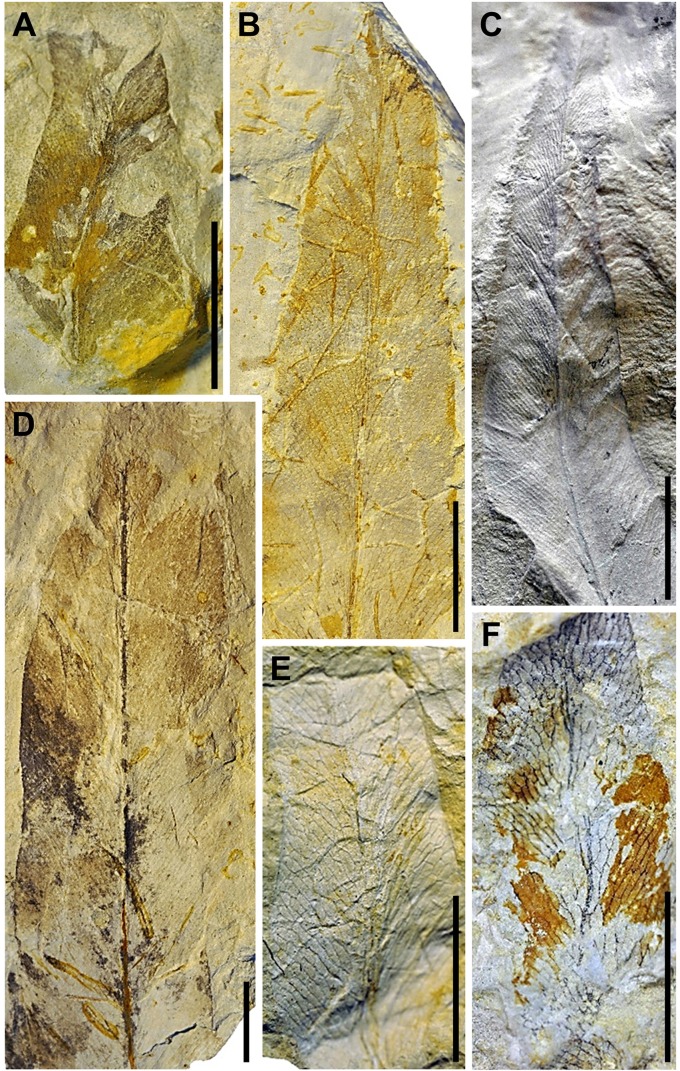
Plant megafossil remains of *Acrostichum lanzaeanum* discovered at the La Val fossil site (II). (A) EMPZ 2016/11-LVNH2-4-1, pinna fragment from level LVNH2. (B) EMPZ 2016/11-LVNH2-9-1, pinna from level LVNH2. (C) EMPZ 2016/11-LVNH2-5-1B, pinna from level LVNH2. (D) EMPZ 2016/11-LVNH2-20-1A, pinna from level LVNH2. (E) EMPZ 2016/11-LVNH-93-1A, pinna from level LVNH. (F) EMPZ 2016/11-LVNH-21-2A, pinna from level LVNH. Scale bars = 1 cm.

Synonymy

**Table:** 

1908	*Chrysodium subhaidingerianum* nov. sp.–Fliche, p. 77–81, pl 1, Figs 1–4.
1931	*Acrostichum (Chrysodium) lanzaeanum* (Vis.) Reid et Chandler–Depape and Bataller, p. 203–204, pl XI, Figs 1–6.
1950	*Acrostichum (Chrysodium) lanzaeanum* (Vis.) Reid et Chandler–Bataller and Depape, p. 10–12, Fig 1A and 1B.
1961	*Acrostichum (Chrysodium) lanzaeanum* Reid et Chandler–Bauzá-Rullán, p. 162–163, Figs 6 and 7.
1965	*Acrostichum* (*Chrysodium*) *lanzeanum* (Vis.) Reid y Chandler–Vicente-Castells, p. 4.
1971	*Acrostichum lanzaeanum* (V.) Reid et Chandler–Fernández-Marrón, p. 11–13, pl 1, Fig 1.
1982	*Acrostichum lanzeanum* (Visiani) Reid y Chandler– Álvarez-Ramis, pl 1, Figs 4 and 5.
1986	*Acrostichum* (*Chrysodium*) *lanzaeanum* (Vis.) Chandl.– Álvarez-Ramis and Ramos-Guerrero, p. 86, Fig 3.
1987	*Acrostichum lanzaeanum* (Reid) Chandler– Álvarez-Ramis et al., p. 350, pl I, Fig 1.
1992	*Acrostichum lanzaeanum* (Visiani) Reid et Chandler–Sanz de Siria, p. 278–279, pl 1, Fig 1.

Description. Pinnae with linear-lanceolate to elliptical shape; maximum preserved length 10–95 mm and width 9–40 mm; the apex is acuminate and acute-angled; the base is not visible; from the thick midvein closely spaced secondary veins arise, initially at an acute angle and then curving at a right angle; the secondary veins anastomose repeatedly, forming numerous rectangular to polygonal areoles (four to five sides); veinlets absent; margin entire.

**Fig 7:**
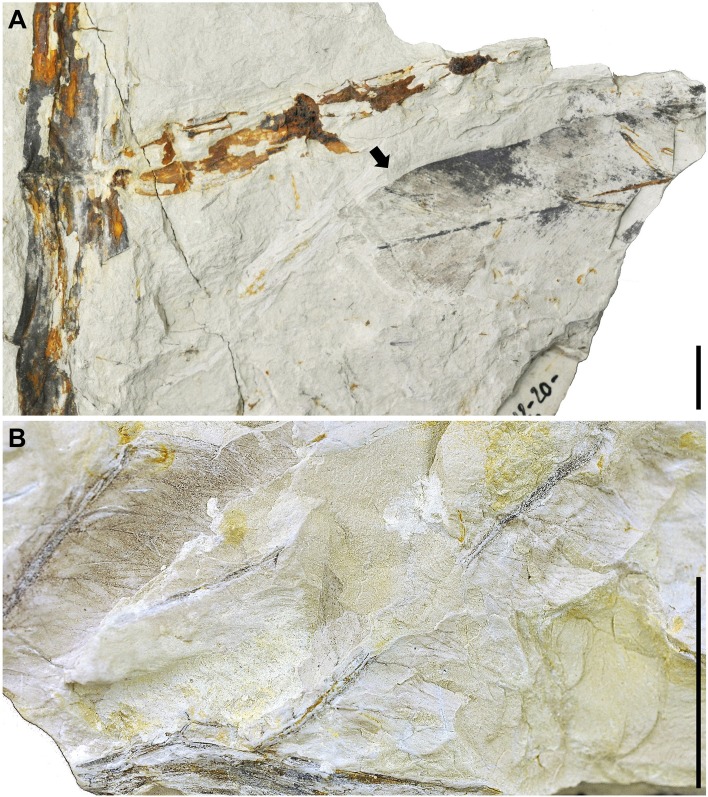
Evidence of associated plant megafossil remains discovered at the La Val fossil site. (A) *Equisetum* stems from level LVNH2 with a fragment of *Acrostichum* pinna at the same level (indicated by arrow). (B) Pinna fragment of *Cyclosorus* from level LVNH. Scale bars = 1 cm.

Remarks. The fossil remains of *A*. *lanzaeanum* appear in levels LV3, LV5, LV6, LVNH and LVNH2 (Fig 2). All of these plant megafossils are preserved as compressions and impressions. Level LV3 corresponds to a fine-grained sandstone bed with scarce root traces. These sediments are interpreted as a crevasse splay deposit. In this bed, the scarce recovered fossil remains of *Acrostichum* correspond to broken pinnae fragments (Fig 5C and 5D). The levels LV5, LV6, LVNH and LVNH2 consist of laminated mudstone beds and exhibit bioturbation and fossil root traces, indicating floodplain areas with pedogenic processes (see S1 and S2 Figs for details). The fossil remains of *Acrostichum* are scarce in levels LV5 and LV6. These remains are however common in the levels LVNH and LVNH2. Interestingly, other associated plant macrofossils also occur in such levels. Stems and rhizome fragments of *Equisetum* are common and usually anatomically-connected in the levels LVNH2 and LVNH pinnae (Fig 7A). Some fragments of pinnae and fronds of fern *Cyclosorus stiriacus* (= *Pronephrium stiriacum*, [17]) from the level LVNH can be also observed (Fig 7B).

Occurrences. In Spain, the oldest macroremains of *Acrostichum lanzaeanum* occur in Bartonian deposits (41.2–37.8 Ma) in Sant Vicenç de Castellet (Barcelona) [21] and at the quarries of Balsamuller and Can Font Els Condals (Manresa) [22]. It is found in early Oligocene deposits (Rupelian, 33.9–28.1 Ma) in Tárrega (Lérida) (e.g., [31–33]), Cervera (Lérida) [33–35], Son Fé Mine (isle of Mallorca) [36] and Peguera (Mallorca) [19]. It also exists in sediments from the late Oligocene (Chattian, 28.1–23.01 Ma) in Son Ferragut (Mallorca) [16]. Some authors (e.g., [33,35]) infer the presence of *Acrostichum* in Mallorca (Burdigalian, 22.44–16.97 Ma) during the Miocene; however, we have strong reservations about this and feel it is not well supported by the evidence (e.g., see [37–38]).

## Discussion

### Paleoenvironment and Paleogeography

The Sariñena Formation is composed of terrigenous rocks, which are considered to have been deposited in a large fluvial system, including alluvial fans (e.g., [26]). Indeed, the identified lithofacies associations (Figs 3 and 4) CS, tSM and chSM correspond to middle (CS) and middle-distal (tSM and chSM) sectors of one of these alluvial fans, called herein the Estadilla fan. These facies represent large polygenic-conglomerate fans where fluvial processes dominated [26]. This type of fan has a large radius and shows a gradual transition in sedimentological processes from proximal to distal areas. In particular, La Val belongs to a distal facies (tSM) which corresponds to the middle-distal portion of the alluvial fan (Figs 3 and 4). The La Val site also corresponds to a retrogradation stage of the alluvial fan (Fig 3), when the decrease in tectonic activity in the area resulted in the retreat of the facies more proximal and allowed the development of the floodplains. These overbank areas were affected during their existence by repeated flooding and drying events, due to the overflow of the surrounding channels when water flowed over the banks and out on to the overbank areas. The presence of numerous crevasse splays and levee deposits in the floodplain indicates the proximity of overbank areas near the active channels. In certain areas, very shallow ponds developed when the water table was close to the surface, with vegetation growing on their shores. These floodplains were affected by numerous pedogenic processes and bioturbation.

During the late Oligocene (Chattian), the paleogeographical reconstruction of the area shows that the alluvial fans retrograded and their characteristic sedimentation included distal alluvial plains and mudflats [26]. The eastern sector was covered by a large alluvial fan (Huesca fan), which entered the basin from the northeast; another large fan developed simultaneously (Balces fan), which entered the basin through a palaeovalley located in the west. This fan was much smaller than the Huesca fan, which collected its waters [26]. The coalescence of both large fans led to the creation of a large fluvial system (Huesca fluvial system, [39]).

### Taphonomy

The presence of anatomically-connected pinnae of ferns suggests a flooding event that likely included river bank collapse [40]. Fern fronds do not abscise naturally but are usually degraded after death, when still attached to the living plant [41]. The existence of entire well-preserved fronds in the water would have only been made possible by river bank collapse, carrying whole plants towards the deposition area (e.g., overbank areas) [40]. So, the presence of fronds of *Acrostichum* and *Cyclosorus* in LVNH implies flooding events with river bank collapse (Figs 5–7). This scenario is also supported by the occurrence in the same bed, of *Cyclosorus*, a fern well known for its ability to colonise disturbed sites such as landslides and roadside banks [6] (Fig 7B). These *Acrostichum* and *Cyclosorus* fronds seem to indicate that they were transported only a minimum distance and, therefore, they are considered as para-autochthonous plant remains [40]. This observation is also supported by the presence of numerous rooting structures in the sampled levels (S1C and S1D, S1F, S2A and S2G Figs). Likewise, despite that the preservational status of *Acrostichum* plant remains is usually as isolated pinnae, i.e., without base and without connection with rachis, the presence also of well-connected pinnae (Fig 5E and 5F) would be also in agreement with a para-autochthonous assemblage.

Then again, the presence of near-complete or entire *Acrostichum* pinnae (e.g., LVNH and LVNH2) indicates only a short transportation by water, and they are also considered as para-autochthonous plant remains (e.g. Figs 6B–6D and 7A). All these para-autochthonous remains suggest that they originally grew close to the deposition areas. On the other hand, the existence of broken pinnae (e.g. Fig 6A) can be explained by a longer water transport, from areas where ferns grew to the deposition areas (e.g., LV3, LV5, LV6, LVNH and LVNH2). Thus, these megafossil remains are considered as allochthonous remains. In the case of anatomically-connected *Equisetum* megafossil remains (e.g. LV6, LVNH2), these are interpreted as having been transported for a very short time by water and, therefore, are also considered as para-autochthonous remains (Fig 7A).

### *Acrostichum*: a pioneering plant of floodplain areas

Floodplains are harsh environments for the colonisation and establishment of plants. In these areas, water directly affects the growing conditions of floodplain vegetation. These plants must resist the sheer stress of flowing water and live with a greater probability of being physically disturbed. Likewise, they are exposed to abrasion and burial from bedload and to physical removal by erosion [42]. Frequent erosional and depositional disturbances from flooding favour pioneering over competitive species and decrease competition for resources. These disturbances tend to decrease the competitive ability of species and thus modify the dominance hierarchy within the community [42,43]. Plants in this community have numerous specific adaptations, e.g., those related to flooding, sediment deposition, physical abrasion and stem breakage [42].

In the La Val fossil site, the watercourses created a mosaic of meandering stream channels and floodplains within the alluvial fan. Overbank areas of La Val were zones affected by numerous and recurrent floods. Flooding mechanically disturbed the vegetation of these areas through erosion of the soil surface and abrasion by transported sediment. These disturbances favoured pioneering species such as *Acrostichum*, *Equisetum* or *Cyclosorus* and the recurrent floods prevented the development of trees and of an overstory. Moreover, the crevasse splay deposits (e.g., LV3) were affected by regular floods, and they were subaerially exposed; these areas were very close to active channels and very well drained. According to Hamer et al. [30], these types of sediments were colonised by herbaceous vegetation.

Typically, crevasse sediments inundate standing vegetation of the back levee, lateral swamps or ponds and floodplains [44]. In these deposits, forest litter can be preserved and mixed with river-transported riparian debris and detritus from the levee community [44]. In LV3 (Fig 2), fossil remains show that *Acrostichum* grew within the levee’s vegetation and/or close to, or on, the river banks. Similarly, some remains were transported by the river from other areas. *Acrostichum* colonises the current levees and is common in wet banks [45–46]. It is also a member of the riparian vegetation, and it grows along and/or next to stream or river margins, especially from the middle to the upper regions of the river [7,9]. In these areas, this fern is an understorey plant, and it grows in the shelter of big plants and/or among small vegetation [45].

The overbank areas of the La Val fossil site (LV5, LV6, LVNH, LVNH2) were affected by regular floods and covered by herbaceous vegetation and/or low stature plants whose rooting systems demonstrate a fluctuating water table [30]. All these areas were near the active channels, well-oxygenated and drained by ground waters that removed the organic matter [30]. However, ephemeral ponds also existed in these floodplain areas (e.g., LVNH and LVNH2). Overall, remains of the interfluve predominate over large areas in the floodplain deposits [44]. While *Acrostichum* remains were transported from nearby areas, it also lived on these floodplain areas, as well as on, or close to, the shores of ephemeral ponds.

Garcia-Massini et al. [6,13] suggest that the abundance of ferns in the same stratum is attributed to their capacity to grow in poorly oxygenated, waterlogged settings, as well as to their ability to be early ecological pioneers, showing a preference for ponded or waterlogged areas. This situation occurs in levels LVNH and LVNH2, among others. The co-existence and abundance of *Equisetum* and *Acrostichum* in the same beds indicates that levels LV5, LV6, LVNH and LVNH2 all have the characteristic of a short-lived inundated terrain (e.g., floodplain area), where the water table was temporarily high, or an ephemeral pond (e.g., LVNH and LVNH2). This suggests the presence of poorly-vegetated wetland environments dominated by pioneering taxa. Moreover, *Acrostichum* is associated with early colonisation events of disturbed environments [6]. The presence of *Equisetum* and *Cyclosorus* also supports this interpretation. Both *Equisetum* and *Cyclosorus* are rapid colonisers of disturbed habitats, and have been found in swampy to marshy, floodplain, and volcanic paleoenvironments in association with other ferns [6]. Current *Equisetum* mainly grows in areas of a high water table (e.g., rivers, streams) not deeper than 0.5 m. Stem bases are immersed in the water in areas where groundwater reaches the surface whereas, in areas of slowly flowing surface water (e.g., flooded areas), rhizomes and roots grow submerged [47].

Jarzen and Dilcher [46] highlight that *Acrostichum* is an aggressive fern, and it tends to become weedy in disturbed sites. Sometimes, this fern is also associated with weedy plants in riparian environments. For instance, Rahman et al. [9] investigated the distribution of riparian corridor plants along the Perai River Estuary (Penang, Malaysia). The upper regions of this corridor were disturbed by human activities (e.g., paddy fields). These regions were occupied by a majority of weed species of angiosperms such as *Cassia tora* Linnaeus (woody shrub), *Eleusine indica* (L.) Gaertner (grass), *Scirpus grossus* Linnaeus (sedge), and *Euphorbia hirta* Linnaeus (broad leaf weed), or ferns such as *Blechnum orientale* Linnaeus and *Lygodium flexuosum* (L.) Swartz. In this community, *Acrostichum* grew associated with these weedy plants in these river regions.

Interestingly, García-Massini et al. [6] describe transient environments colonised by pioneer vegetation in a late Oligocene succession of volcaniclastic deposits from the northwestern Ethiopian Plateau (Chilga strata unit). This Chilga plant diversity was dominated by ferns; amongst which, being especially relevant here, were *Acrostichum*, *Equisetum* and *Cyclosorus*. A few angiosperm taxa typical of disturbed environments (e.g., *Typha*, *Pandanites* and *Hyphaene*) were also documented. The Chilga strata corresponded to overbank areas with ephemeral ponds and small channels with crevasse deposits. Physiographic changes of the Chilga paleoenvironment were interpreted to have resulted directly from the influence of volcanism on the surface environment [6]. So, despite the fact that the sedimentological environment of the La Val site and Chilga strata are similar, their physiographic history is different. While in Chilga strata, the environment changed mainly by airfall ash and ephemeral discharge of sediments by braided streams [6], the environmental changes in the La Val floodplains were produced by several floods that affected the vegetation. Both paleoenvironments were colonised by similar plant communities.

## Conclusions

The late Oligocene La Val fossil site (northeastern Iberian Peninsula) represents a continental fluvial paleoenvironment that was fully isolated from marine influence. The streams and/or rivers created a mosaic of meandering stream or river channels and floodplains within an alluvial fan. From this outcrop, reported fossil evidence of *Acrostichum lanzaeanum* reveals that this fern grew within the levee’s vegetation or close to the river banks next to the shores of ephemeral ponds. The co-existence of *Equisetum* and *Acrostichum* in assemblages suggests the presence of short-lived inundated terrain, such as either a floodplain, where the water table was temporarily high, or an ephemeral pond. This scenario indicates, in addition, poorly-vegetated wetland environments dominated by pioneering taxa. The La Val plant community is similar to that of Chilga strata during the late Oligocene. However, the environmental changes registered in the La Val floodplains suggest the presence of recurrent floods. In both cases, evidence shows that *Acrostichum* grew and developed as a coloniser plant in disturbed areas.

## Supporting Information

S1 Fig Sedimentary structures observed in the La Val fossil site (I). (A) Floodplain deposits close to level LV2: Horizontal lamination (black arrow) and mottling (white arrow). (B) Floodplain deposits close to level LV2: Ripple-marks (black arrows). (C) Floodplain deposits at level LV6: Horizontal lamination (black arrows) and small roots (white arrows). (D) Floodplain deposits at level LV5: A fossilized root (black arrow). (E) Floodplain deposits between the levels LV4-5: Mud-cracks (black arrows). (F) Floodplain deposits at level LV4: Horizontal lamination (black arrow), mottling (white arrow) and root (grey arrow). (G) Crevasse deposits at level LV7: Ripple-marks (black arrows) and horizontal lamination (white arrow). Scale bars = 2 cm.
(TIF)

S2 Fig Sedimentary structures observed in the La Val fossil site (II). (A) Floodplain deposits at level LV5: small root (black arrow), scale bar = 2 cm; (B) Floodplain deposits: Heterolitic lamination, sand beds (black arrow), mud beds (white arrow), scale bar = 2 cm. (C, D) Floodplain deposits at level LV6: Bioturbation (black arrows), scale bar = 2 cm. (E) Crevasse deposits: Cross lamination (black arrow), scale bar = 10 cm. (F) Crevasse/levee deposits: scour fill deposits (black arrow), scale bar = 2 cm. (G) Floodplain deposits at level LVNH2: a perpendicular section of small roots (black arrows), and mottling (white arrow), scale bar = 2 cm.
(TIF)

S1 Table Measurements data of fossil specimens of *Acrostichum* collected from the La Val fossil site. (PDF)
