# Supplementary figures and images for: Acrostichum, a Pioneering Fern of Floodplain Areas from the Late Oligocene Sariñena Formation of the Iberian Peninsula
Source: PLoS One. 2016 Sep 15;11(9):e0162334. doi: 10.1371/journal.pone.0162334 (PMC5024994; doi:10.1371/journal.pone.0162334)

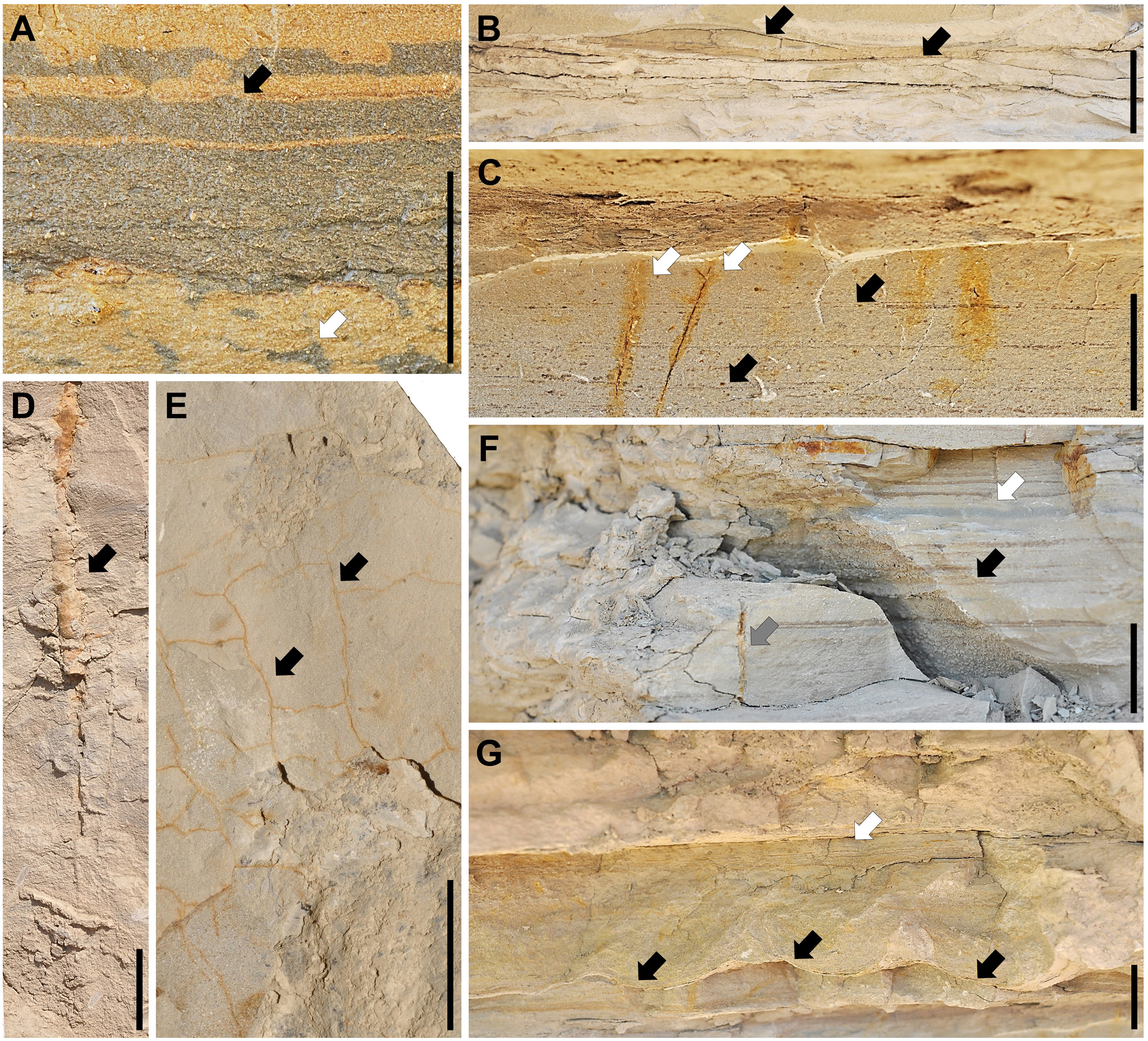

Supplement: S1 Fig — (A) Floodplain deposits close to level LV2: Horizontal lamination (black arrow) and mottling (white arrow). (B) Floodplain deposits close to level LV2: Ripple-marks (black arrows). (C) Floodplain deposits at level LV6: Horizontal lamination (black arrows) and small roots (white arrows). (D) Floodplain deposits at level LV5: A fossilized root (black arrow). (E) Floodplain deposits between the levels LV4-5: Mud-cracks (black arrows). (F) Floodplain deposits at level LV4: Horizontal lamination (black arrow), mottling (white arrow) and root (grey arrow). (G) Crevasse deposits at level LV7: Ripple-marks (black arrows) and horizontal lamination (white arrow). Scale bars = 2 cm. (TIF) [file pone.0162334.s001.tif]

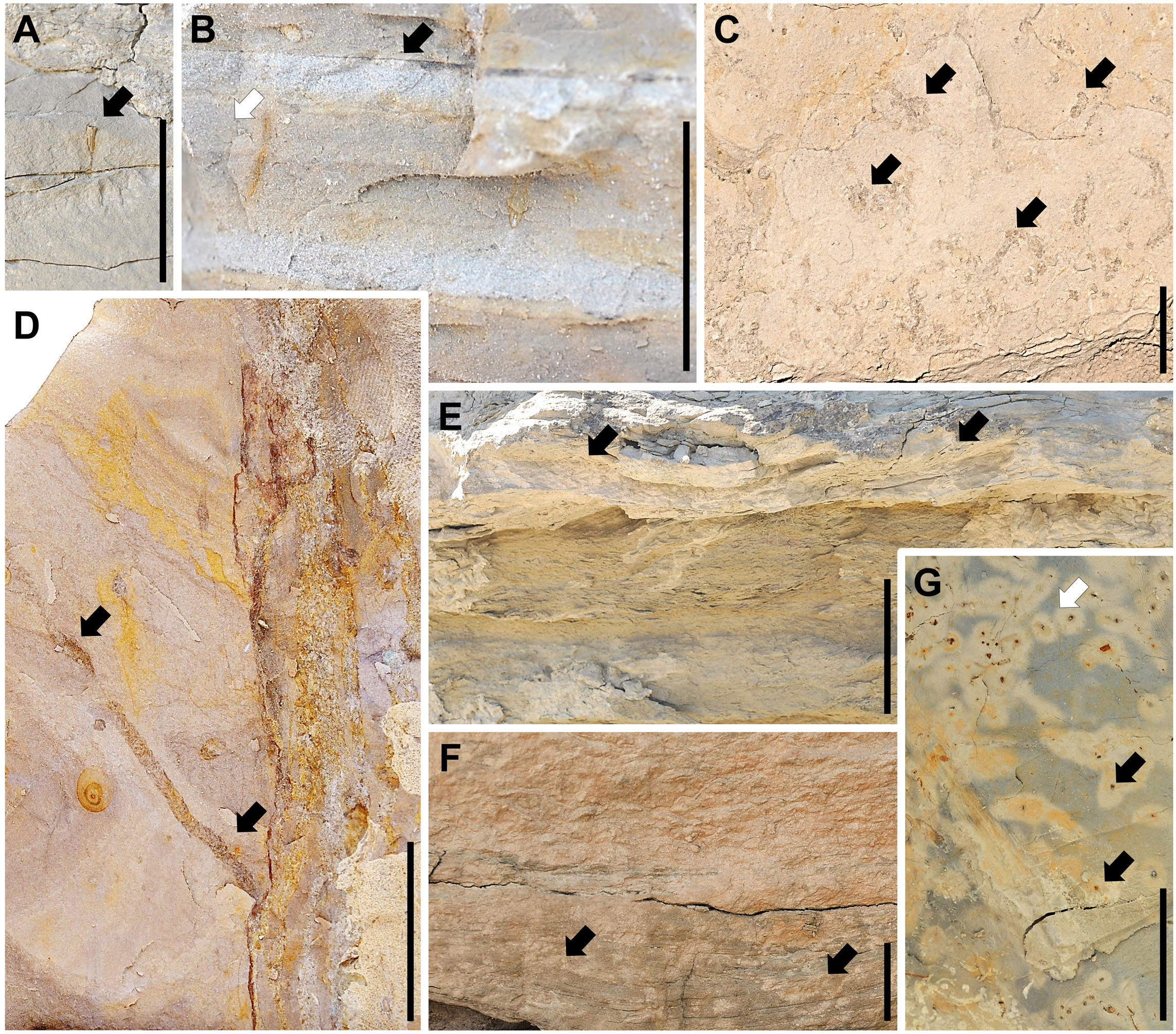

Supplement: S2 Fig — (A) Floodplain deposits at level LV5: small root (black arrow), scale bar = 2 cm; (B) Floodplain deposits: Heterolitic lamination, sand beds (black arrow), mud beds (white arrow), scale bar = 2 cm. (C, D) Floodplain deposits at level LV6: Bioturbation (black arrows), scale bar = 2 cm. (E) Crevasse deposits: Cross lamination (black arrow), scale bar = 10 cm. (F) Crevasse/levee deposits: scour fill deposits (black arrow), scale bar = 2 cm. (G) Floodplain deposits at level LVNH2: a perpendicular section of small roots (black arrows), and mottling (white arrow), scale bar = 2 cm. (TIF) [file pone.0162334.s002.tif]
