# Supplementary material for: Acrostichum, a Pioneering Fern of Floodplain Areas from the Late Oligocene Sariñena Formation of the Iberian Peninsula
Source: PLoS One. 2016 Sep 15;11(9):e0162334. doi: 10.1371/journal.pone.0162334 (PMC5024994; doi:10.1371/journal.pone.0162334)
Supplement: S1 Table — (PDF) [file pone.0162334.s003.pdf]

S1 Table. Measurements data of fossil specimens of *Acrostichum* collected from the La Val fossil site.

SPECIMEN	Width (mm)	Lenght (mm)
EMPZ 2016/11-LV5-33-1A/1B	18	67
EMPZ 2016/11-LV5-36-1	25	60
EMPZ 2016/11-LV5-37-1	20	56
EMPZ 2016/11-LV5-38-1	17	70
EMPZ 2016/11-LV5-39-1	20	93
EMPZ 2016/11-LV5-40-1	20	95
EMPZ 2016/11-LV6-84-1A/1B	15	55
EMPZ 2016/11-LVNH-21-2A/2B	9	18
EMPZ 2016/11-LVNH-80-1	15	32
EMPZ 2016/11-LVNH-81-1A/1B	16	56
EMPZ 2016/11-LVNH-86-1A/1B	18	30
EMPZ 2016/11-LVNH-89-1	13	30
EMPZ 2016/11-LVNH-91-1A/1B	14	36
EMPZ 2016/11-LVNH-93-1A/1B	19	10
EMPZ 2016/11-LVNH-93-2A/2B	17	18
EMPZ 2016/11-LVNH2-3-1A/1B	20	40
EMPZ 2016/11-LVNH2-5-1A/1B	14	53
EMPZ 2016/11-LVNH2-5-2	25	54
EMPZ 2016/11-LVNH2-8-1	12	72
EMPZ 2016/11-LVNH2-9-1	27	52
EMPZ 2016/11-LVNH2-12-1	40	51
EMPZ 2016/11-LVNH2-13-1	26	60
EMPZ 2016/11-LVNH2-14-1	24	80
EMPZ 2016/11-LVNH2-16-1	30	80
EMPZ 2016/11-LVNH2-17-1	36	50
EMPZ 2016/11-LVNH2-17-2	18	50
EMPZ 2016/11-LVNH2-18-1A/1B	27	78
EMPZ 2016/11-LVNH2-19-2	33	75
EMPZ 2016/11-LVNH2-20-1A/1B	25	75
